# Exposure characteristics and risk assessment of air particles in a Chinese hotel kitchen

**DOI:** 10.3389/fpubh.2022.1019563

**Published:** 2022-10-25

**Authors:** Zanrong Zhou, Xiangjing Gao, Yiyao Cao, Hua Zou, Yulan Jin

**Affiliations:** ^1^Institute of Occupational Health and Radiation Protection, Zhejiang Center for Disease Control and Prevention, Hangzhou, China; ^2^Hangzhou Hospital of Zhejiang Medical and Health Group, Hangzhou, China

**Keywords:** particles, quantity concentration, mass concentration, surface area concentration, risk assessment

## Abstract

**Background:**

The hazards of kitchen particles have attracted social attention, but their distribution characteristics and risk assessment are rarely reported.

**Objective:**

To explore the temporal and spatial distribution characteristics of kitchen particles, analyze the variations in characteristics of number concentration (NC), mass concentration (MC), surface area concentration (SAC), and particle size distribution, provide reference indexes for evaluating worker exposure, evaluate the risk of kitchen particles, as well as suggest improvements and control measures.

**Patients and methods:**

Different cooking posts in a Chinese hotel kitchen were selected to monitor exposure to particles, explore the temporal and spatial distribution characteristics of NC, MC, and SAC of particles in the cooking post, analyze changes in the particle size, compare the individual exposure of particles between the cooking and steaming posts, and analyze the correlation between NC, MC, and SAC. Risk assessment of kitchen ultrafine particles was performed using a Nanotool.

**Results:**

The sizes and fluctuation ranges of NC_10 − 500*nm*_ at cooking posts during lunch preparation and at peak periods were significantly higher than those at the end of the lunch period. The mean values of MC_10 − 500*nm*_ during the lunch preparation peak and ending periods were 0.149, 0.229, and 0.151 mg m^−3^, respectively. The mean values of SAC_10 − 500*nm*_ were 225, 961, and 466 μm^2^·cm^−3^, respectively. The mode diameter of exposed particles at the cooking post [(34.98 ± 2.33) nm] was higher than that at the steaming post [(30.11 ± 2.17) nm] (*P* < 0.01). The correlation between SAC_10 − 500*nm*_ and NC_10 − 500*nm*_ (*r* = 0.703) was the strongest. Nanotool gave a hazard rating ratio, exposure rating ratio, and risk ratio of 0.75.

**Conclusion:**

The sizes of the NC, MC, and SAC of the particles at the cooking post were related to the kitchen operations. Since kitchen particles are of high exposure and risk levels, protective measures should be formulated and implemented to deal with them safely.

## Introduction

Catering-related kitchen fume pollution is increasing in severity. It is now one of the main air pollutants in indoor living environments. Kitchen oil fumes are aerosols composed of gas, solids, and liquids (1), which can float in the air for a long time, and contain many ultrafine particles smaller than 0.1 μm. Ultrafine particles are generally defined as aerodynamic, geometric, or having migration diameters < 100 nm, and are marked by particularity, diversity, and potential harm (2). The new physical and chemical characteristics of ultrafine particles lead to complex exposure characteristics and different biological effects (3). Results from *in vitro* and animal experiments have shown that ultrafine particles have greater toxicological effects than large particles of parent materials (4). The health risks caused by the characteristics and widespread existence of ultrafine particles have attracted extensive attention, but there is a lack of population exposure data. The direct reason is that there is no perfect method for the exposure assessment of ultrafine particles in China or elsewhere. One reason for the lack of exposure assessment methods is that people do not know much about the exposure characteristics of ultrafine particles in the workplace. This paper mainly studied the distribution characteristics of particulate matter in Chinese kitchens, the relationship between Sac, Mc and Nc, and risk assessment methods, focusing on the relationship between SAC, MC and NC, which is the difference between related studies (5).

Currently, there is not much literature in China and worldwide on the harm of kitchen oil fume particles to the human body and how to control the amount of oil fume particles (6, 7). Studies have shown that kitchen oil fume particles can cause cardiovascular and cerebrovascular diseases, cancer, skin damage, respiratory diseases and so on (8). Researchers have reported the induced damage of kitchen oil fume particles to DNA (9), the source of indoor ultrafine particles (10), the differences in the number of particles produced by different cooking methods (11), the impact of different energy heating methods on particles in the kitchen (12), and differences in the number of particles produced by variations in oil heating. Few studies have examined the distribution and exposure characteristics of oil fume particles and assessed the risks of kitchen particles. However, assessment methods and improvements that should be made and adopted still need to be discussed. Similarly, due to their unique nature and specific size, these particles may cause different health hazards than other dust materials. Therefore, the method for evaluating their exposure concentrations and risk should be different from that for other dust material counterparts. The toxicity of ultrafine particles may be due to their small size, high surface activity, charge, and dissolution rate. Kitchen particles have high surface activity, which can promote the ability of nanoparticles to enter cells, resulting in damage to the cells, proteins, and genes in the lungs, as well as the cardiovascular and nervous systems (13). Kitchen oil fumes contain a large number of free radicals with large molecular weight and high stability, which can generate reactive oxygen free radicals and lipid peroxides when entering the body, an important cause of lung cancer, tracheitis, pneumonia, and emphysema (14).

Thus far, the existing studies cannot clarify their damage to the body. Moreover, the international standard of exposure limit has not yet been determined. Due to the potential toxic effect of kitchen particles on human health, it is very important to conduct a risk assessment of these particles. The impact of kitchen particles on human health is a research field worthy of discussion. Thus, we used existing data to evaluate the risk of occupational exposure to kitchen particles, as well as to establish a comprehensive and systematic kitchen particles database. This study examined the number concentration (NC), mass concentration (MC), surface area concentration (SAC), and size distribution of particles in a Chinese kitchen. It also explored the temporal and spatial distribution characteristics of each index and focusing on the correlations between MC, NC, and SAC, which is the difference between related studies (5). This study is expected to preliminarily clarify the exposure characteristics of particles in kitchen fumes, suggest better indicators for the exposure assessment of kitchen workers, provide a basis for the health risk management of exposed people, and lay an experimental foundation for future studies on the health effects of kitchen fumes.

## Materials and methods

### Kitchen selection

A Chinese hotel kitchen was selected as the survey site. The kitchen is set on the second floor of the north side of the hotel, which connects to the outside world only by the smoke exhaust duct. The kitchen has an area of 5 m × 16 m, and is divided into a storage room, preparation area, and cooking area. There were vegetables, frozen food, and other food materials in the storage room, from which they were taken out by the food preparation personnel in that order during preparation. The preparation area was the operation area for cold dish preparation, cleaning, and chopping food materials. The cooking area was an operation area for cooking food materials. The hood ventilation facilities were set above the front of the five cooking stoves in the operation area, but there was only one hood ventilation system in this kitchen. During operation, the air velocity at the capture point was about 0.8 m · s^−1^. The chef did not wear protective masks or noise-proof earplugs, and lunchtime was mainly from 10 a.m. to 2 p.m. The preparation area was adjacent to the cooking area. Therefore, both work areas were exposed to the oil fumes generated during cooking.

### Measurement indicators and instruments

The measurement indices were divided into exposure concentration and particle size distribution. The exposure concentration indices were MC, NC, and SAC (referring to the sum of all particle surface areas in unit volume), and the particle size distribution index was the particle size distribution of NC. In addition, the auxiliary measurement indicators include air temperature, air pressure and wind speed in meteorological conditions. Table 1 shows the main instruments and parameters. The instruments used included the aerosol monitor Dusttrak 8533, the ultrafine particle counter 3007, the nanoparticle aerosol monitor Aero Trak 9000, the scanning electromigration particle size meter SMPS 3034, the optical particle sizer OPS 3330, the meteorological condition meter 9565 (TSI, USA), and the nanoparticle analyzer Discmini (Testo, range 10–700 nm, Germany). All instruments are returned annually to the original factory for calibration.

**Table 1:** The main instruments and parameters.

**Exposure metrics**	**Instruments**	**Particle sizes (nm)**	**Measuring range**	**Sampling rate (L·min^–1^)**	**Log interval (min)**
Total NC	3007 (TSI, USA)	10–1,000	0–100,000 particles·cm^−3^ (pt·cm^−3^)	0.1	1
Personal NC	DiSCmini (TESTO, Germany)	< 700	0–5,000,000 pt·cm^−3^	1.0	1
Total respirable MC	Dust Trak 8533 (TSI, USA)	100–1,000	0.01–150 mg·m^−3^	3	1
SAC	Aero TrakTM 9000 (TSI, USA)	10–1,000	1–10,000 μm^2^·cm^−3^	2.5	1
Size distribution by number	SMPS 3034 (TSI, USA)	10–487	1–2.4 × 10^6^ pt·cm^−3^	1.0	3
	OPS 3330 (TSI, USA)	300–10,000	0–3,000 pt·cm^−3^	1.0	1

### Sampling scheme

**Figure 1:**
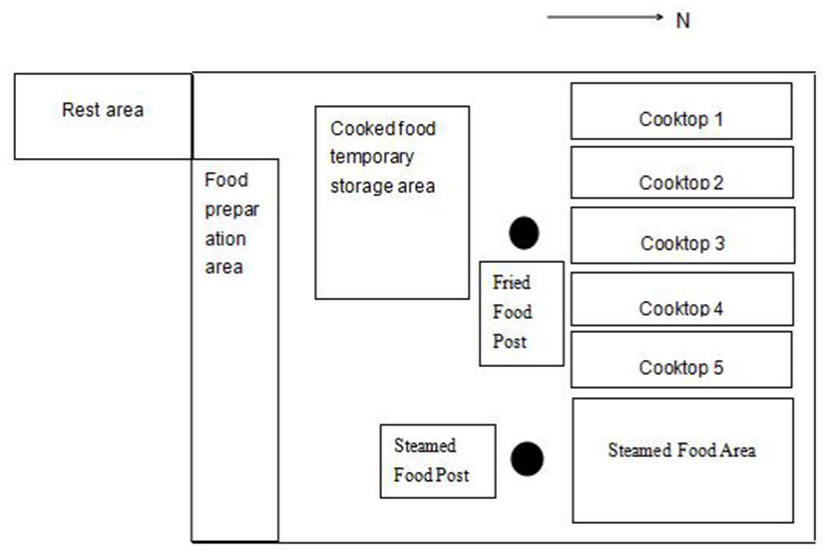
Locations of monitoring sites. •: Detection point.

Firstly, through on-site investigation and pre-detection of the workplace with 3007, the emission source of particles at the detection posts was determined, as well as the sampling time. The specific detection scheme was as follows: (15) ① Background concentration measurement: the concentration of particles in the air in the kitchen between 9 and 9:59 am before cooking on the same day was selected as the background concentration. When detecting the background concentration, there were no workers or other particle release sources. ② Particle detection based on operation activities: the detection location was based on the early field investigation data and pre-detection, and the influences of the chef's operation mode, instruments, and equipment on the chef's operation were considered. The main cooking methods included stir-frying, pan-frying, and deep-frying, which were generally operate under rapid high-heat conditions. There were also steaming posts, which were usually operated under continuous heating. The combination of fixed-point sampling and individual sampling was adopted within the same day at different times, and individual samples were collected for different indicators. The temporal and spatial distributions of particles in cooking posts were analyzed by fixed-point sampling (180 groups of data were collected throughout the preparation, peak, and ending periods). An individual sampling method was used to analyze the particle exposure characteristics of different posts (133 groups of data were collected during the preparation and peak periods). For individual sampling, the instrument was hung on the chef, and the sampling air inlet was clamped at the breathing belt position on the chef's collar. During fixed-point sampling, the sampling and testing instrument was placed at the downwind side of the testing post. The instrument and equipment were as close to the chef as possible without affecting his operation. The point distribution position is shown in Figure 1. The detection height was the worker's respiratory belt level. The detection time was from the beginning of preparation to the end of operation activities. The detection period was 10:45–13:44. Simultaneously, the background value in the kitchen during non-working hours before lunch was detected, and the detection period was 9:00–9:59. Table 2 illustrates the detailed events of each stage.

**Table 2:** Record of activities in each lunch period.

**Activity period**	**Time**	**Activity**
Before lunch	9:00-−9:59	No operation
Lunch preparation	10:45-−11:44	Opening and closing of furnaces 1 and 2; 3 times each
Lunch peak	11:45-−12:44	Furnace 1 was opened and closed 5 times, furnace 2 was opened and closed 3 times, furnace 3 was opened and closed 3 times, furnace 4 was opened and closed 5 times, and furnace 5 was opened and closed 3 times
Lunch closing	12:45-−13:44	No operation

### Risk assessment method

According to previous studies (16), the Nanotool (http://www.controlbanding.net/) is suitable for nanoparticles and has comprehensive advantages in risk assessment; thus, it was selected for use in this study. It was developed by Paik and Zalk at Lawrence Livermore National Laboratory in the United States. The Nanotool uses a scoring system to allocate risk and exposure levels, as well as combines the risk and exposure levels to obtain the risk level in the two-dimensional decision matrix, followed by dividing them into four levels on average. Table 3 shows the hazard and exposure input parameters. Hazards were determined based on particle shape, concentration, surface activity, and toxicity (including carcinogenicity, mutagenicity, and reproductive toxicity). Exposure levels were determined by substance emission potential, activity emission potential, and exposure control.

**Table 3:** Hazard parameters and exposure scenario parameters of oil fume.

**Input information**	**Required information**	**Oil fume**
Nanotool base metal hazard classification input parameters	Carcinogen	Yes
	Reproductive harm	No
	Mutagen	Yes
	Skin hazards	No
	Sensitization	No
Nanotool - nanomaterial hazard classification input parameters	Surface reactivity	Unknown
	Particle shape	Anisotropy
	Particle size	11–40 nm
	Solubility	Insoluble
	Carcinogen	Yes
	Reproductive harm	Yes
	Mutagen	Yes
	Skin hazards	No
	Sensitization	No
Exposure classification input parameters	Aerosol concentration	11–100 mg
	Current engineering control	Local exhaust ventilation
	Number of employees with similar exposures	8
	Operating frequency (year)	Everyday
	Operating time	>4 h

### Statistical analysis

The comparison of NC and the background concentration of particles exposed to different cooking posts and the comparison of the SAC of particles under different modes were analyzed using one-way ANOVAs. The pairwise comparison was performed using the Least-SignificantDifference (LSD)method when the variances were homogeneous. The pattern diameter of particles, or the particle diameter corresponding to the maximum NC of the particles at lunch peak, exposed to different cooking posts was analyzed using a one-way ANOVA with repeated data. The Pearson correlation method was used to analyze the correlations between NC, MC, and SAC. The significance level was set at α = 0.05.

## Results

### Time-concentration particle changes at the fried food post

**Figure 2:**
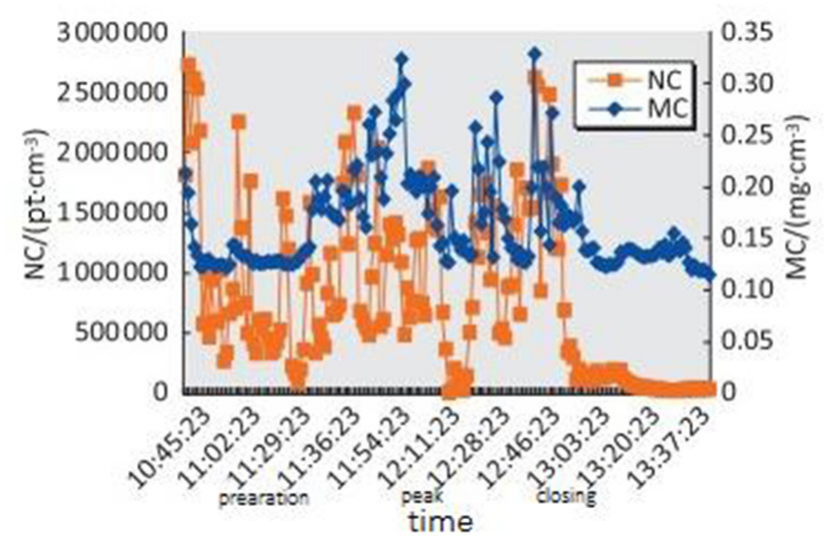
Distribution of NC_10 − 500*nm*_ and MC_10 − 500*nm*_ of fried food posts in the Chinese kitchen in different time periods.

As shown in Figures 2, **4**, the sizes and fluctuation ranges of NC_10 − 500*nm*_ during the lunch preparation and peak periods were significantly higher than those during the lunch ending period. These were related to operational activities. The lunch preparation period was 10:45–11:44, which mainly focused on vegetable preparation, as well as cooking and heating preparation, with no cooking activities. The average NC_10 − 500*nm*_ particle value was ~10^6^·pt·cm^−3^. The lunch peak period was 11:45–12:44. Cooking activities were frequent during this stage, and the average NC_10 − 500*nm*_ value was about 9.8 × 10^5^ ·pt·cm^−3^. There was no significant difference between lunch preparation and lunch peak NC_10 − 500*nm*_. Lunch ended at 12:45–13:44. There were a few cooking activities from 12:45 to 13:00, after which there were none. The average value of NC_10 − 500*nm*_ was 4.2 × 10^5^·pt·cm^−3^, which was lower than that during the preparation and peak period (*P* < 0.01), but higher than the background value (about 0.4 × 10^5^ ·pt·cm^−3^; *P* < 0.01).

MC_10 − 500*nm*_ fluctuated less than NC_10 − 500*nm*_ throughout the whole process. The mean values of MC_10 − 500*nm*_ during the lunch preparation, peak, and closing periods were 0.149, 0.229, and 0.151mg · m^−3^, respectively. MC_10 − 500*nm*_ in the peak period was higher than that in the preparation and closing periods (*P* < 0.05); however, there was no difference in MC_10 − 500*nm*_ between the lunch preparation and closing periods (*P* > 0.05).

**Figure 3:**
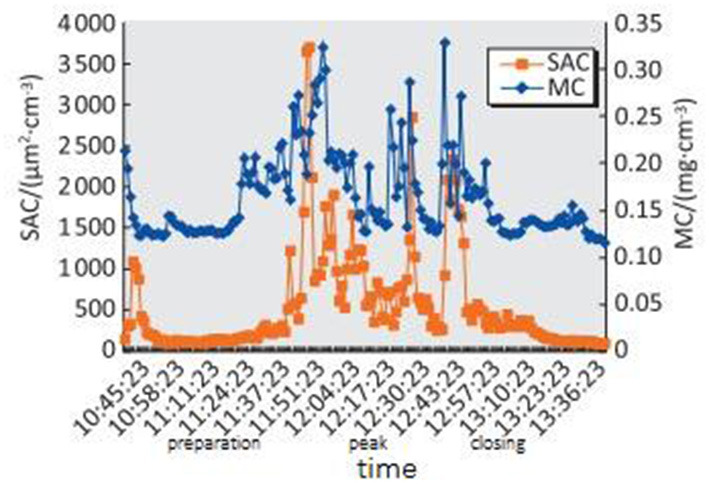
Distribution of SAC_10~500*nm*_ and MC_10~500*nm*_ of particles at fried food posts in the Chinese kitchen in different time periods.

**Figure 4:**
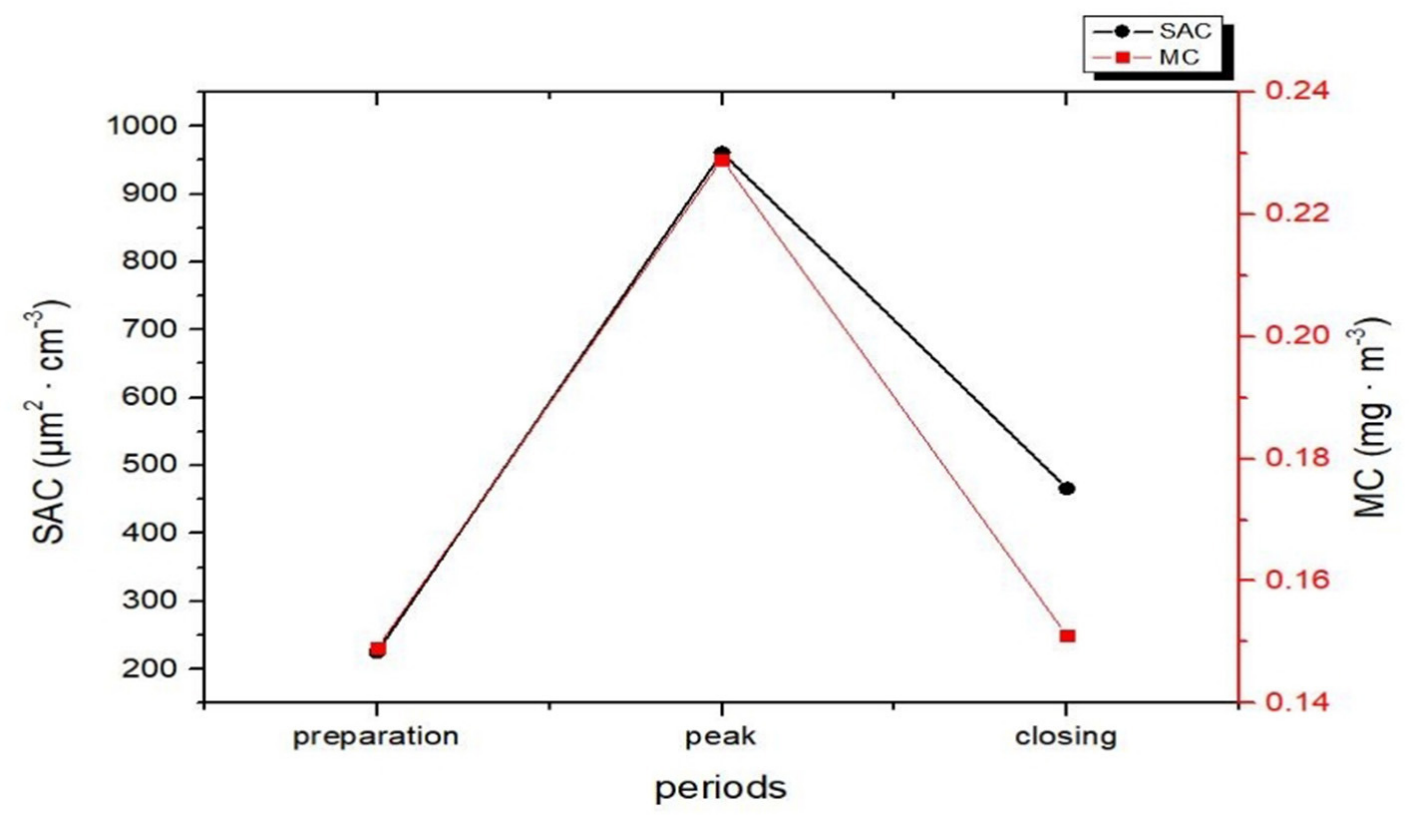
Mean values of SAC_10~500*nm*_ and MC_10~500*nm*_ of particles at fried food posts in the Chinese kitchen in different periods.

As shown in Figures 3, 4, the mean values of SAC_10 − 500*nm*_ during the lunch preparation, peak, and closing periods were 225, 961, and 466 μm^2^ · cm^−3^, respectively. The SAC_10 − 500*nm*_ value was higher during the peak period than during the preparation and ending periods and was higher during the ending period than during the preparation period (*P* < 0.05).

### Particle size characteristics for the fried food posts

**Figure 5:**
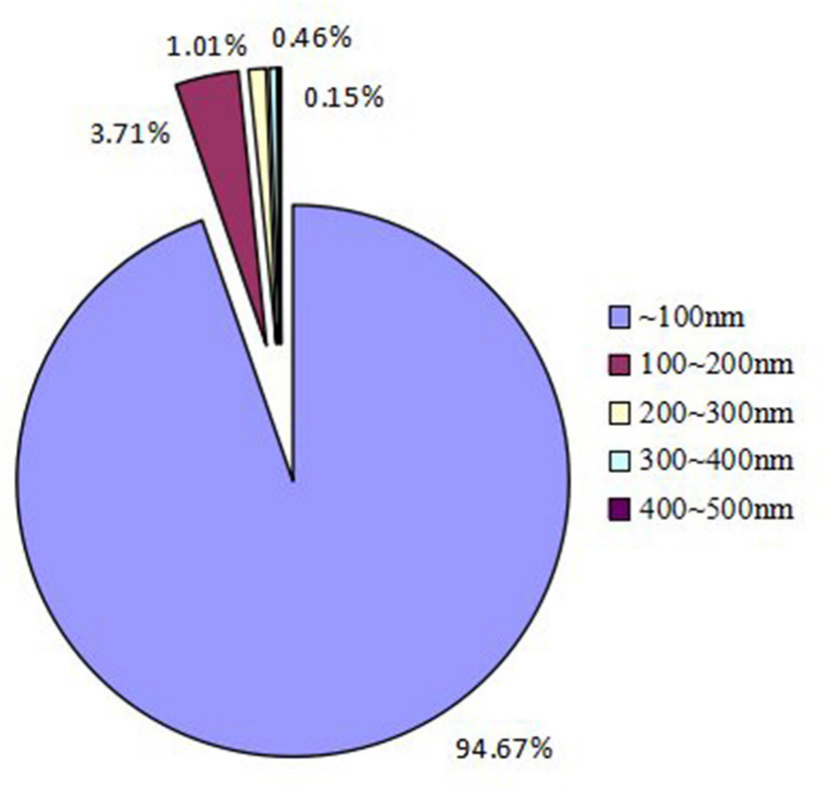
Percentages of particle number distribution with different particle size at lunch peak.

During the lunch peak, the number of particles was distributed from large to small, as shown in Figure 5: within 100nm, > 100–200nm, > 200–300nm, > 300–400nm, and > 400–500nm. The proportion of particles with a particle size less than 100nm and less than 200nm was 94.67% and 98.38%, respectively. When the particle size was less than 200 nm, the NC of the particles of 19nm was the largest, so the mode diameter at the lunch peak was 19nm.

### Individual particle exposure levels at different cooking posts

The NC_10 − 700*nm*_ [(1,032,352 ± 158,231), (668,771 ± 23,623) pt·cm^−3^] of the particles exposed to the cooking and steaming posts was higher than the background value (42,485 pt·cm^−3^) (*P* < 0.01). The NC_10 − 700*nm*_ of the cooking posts was higher than that of the steaming posts (*P* < 0.01), and the mode diameter of the particles exposed to the cooking posts [(34.98 ± 2.33) nm] was higher than that of the steaming posts [(30.11 ± 2.17) nm] (*P* < 0.01), as shown in Table 4.

**Table 4:** Analysis of variance of NC_20 − 700*nm*_ and mode diameter of particles in different posts (*n* = 133).

**Post**	**NC_20–700nm_ size (nm)**	**Mode**
Fried food post (*n* = 133)	1,032,352 ± 158,231^1^^,^ ^2^	34.98 ± 2.33^3^
Steamed food post (*n* = 133)	668,771 ± 23,623^2^	30.11 ± 2.17
Background values	42,485	

1 P < 0.01, compared with steaming posts.

2 P < 0.01, compared with the background values.

3 P < 0.01, compared with steaming posts.

### Correlations between MC, NC, and SAC

The correlation between NC_10 − 500*nm*_ and SAC_10 − 500*nm*_ was the strongest, with a correlation coefficient of 0.703 (*P* < 0.01). The correlation coefficients of NC_10 − 500*nm*_ and MC_10 − 500*nm*_, as well as SAC_10 − 500*nm*_ and MC_10 − 500*nm*_ were 0.412 and 0.351, respectively (*P* < 0.05), as shown in Table 5.

**Table 5:** Correlation Analysis between MC_10 − 500*nm*_, NC_10 − 500*nm*_ and SAC_10 − 500*nm*_ (*n* = 180).

**Index**	**SAC_10–500nm_**	**NC_10–500nm_**	**MC_10–500nm_**
SAC_10 − 500*nm*_ (μm^2^/cm^3^)	1.00	0.703^1^	0.351^2^
NC_10 − 500*nm*_ ( × 10^4^p/cm^3^)	–	1.00	0.412^a^
MC_10 − 500*nm*_ (mg/m^3^)	–	–	1.00

1 P < 0.01.

2 P < 0.05.

### Risk assessment of kitchen particles and control measures to be improved

Table 6 shows the risk assessment results of the control classification tool and the preventive measures recommended by the National Institute for Occupational Safety and Health (NIOSH) regulations. Nanotool's hazard and exposure level ratios were 0.75, and the risk ratio was 0.75, suggesting high exposure and risk levels and indicating that protective measures should be formulated and implemented for kitchen oil fumes. According to NIOSH regulations, the prevention and control of ultrafine particles include five elimination steps: replacement, engineering control, administrative control, and personal protective equipment (PPE). Since it is impossible to eliminate and substitute ultrafine particles during cooking, the following control levels should be adopted: engineering control, administrative control, and PPE. Table 5 lists the current control measures and other control measures that need improvement.

**Table 6:** Risk assessment and control measures.

**Tool**	**Hazard level ratio**	**Exposure grade ratio**	**RR**	**Existing control measures**	**Other control measures to be improved**
Nanotool	0.75	0.75	0.75	(1) Engineering control: the gas stove is equipped with LEV (2) Occupational health management system: regular occupational health training, reduce exposure time and conduct occupational health examination for workers. The preventive maintenance plan for ultrafine particles is missing	(1) Engineering control: reasonably arrange the position of air supply and exhaust outlets, distribute air volume, and select the form of air outlet. It is necessary to increase the exhaust speed of LEV (2) Occupational health management system: LEV regular maintenance and inspection plan shall be formulated to ensure the effectiveness of engineering control measures (3) PPE: NIOSH certified N95 or P100 filter mask respirator shall be used, and regular inspection shall be conducted to ensure that workers wear PPE correctly

## Discussion

The NC_10 − 500*nm*_ values and particle fluctuations during the lunch preparation and peak periods were greater than those of the background value. However, there was no difference between the preparation and the peak periods. The kitchen operation during preparation is mainly the pretreatment of some dishes, and a small amount of cooking operation will also produce soot particles. The above factors will increase the exposure of NC_10 − 500*nm*_ during lunch preparation (17).

SAC_10 − 500*nm*_ was different in the three lunch periods, with peak period > ending period > preparation (*P* < 0.05). The particle SAC_10 − 500*nm*_ in the peak period of operation was higher than that of the preparation and ending periods, which was related to the particle NC_10 − 500*nm*_ in the peak period. Thus, the value in the ending period was higher than that in the preparation period, which may be related to the presence of many particles floating in the air during the ending period (18). This study also found a correlation between SAC_10 − 500*nm*_ and MC_10 − 500*nm*_, consistent with the results from a study by Zou et al. (19).

The focus of this study was mainly ultrafine particles within the 100 nm limit. When analyzing the composition of particles according to size, we also analyzed particles with other sizes. Particles above 500 nm have a short residence time in the air and low concentration, which is difficult for the sampling instrument to capture. Regarding composition, the number of particles < 100 nm accounted for over 94%, occupying an absolute advantage, which may be related to the agglomeration effect of ultrafine particles (20).

When studying the NC_10 − 700*nm*_ exposure characteristics of individual particles at the cooking and steaming posts, we found that the NC_10 − 700*nm*_ exposure values of the two posts were both statistically significant according to a one-way ANOVA (*P* < 0.01). Considering that the components of particles in contact with the cooking and steaming posts were not the same, it can be inferred that the cooking post was mainly exposed to a large amount of grease. In contrast, the steaming post was exposed to a large amount of steam. Therefore, the harm of particle exposure at the cooking post may be much greater than that at the steaming post.

Correlation analysis showed that the correlation between SAC_10 − 500*nm*_ and NC_10 − 500*nm*_ was higher than that between SAC_10 − 500*nm*_ and MC_10 − 500*nm*_, which is consistent with the results of a study by Heitbrink et al. (21). Toxicological studies have also shown a strong dose-response relationship between the surface area dose of very low solubility fine particles and ultrafine particles and inflammatory lung response (22). Moreover, epidemiological studies have shown a correlation between SAC and population health risk (23). Further, there was no linear correlation between the measurement results of daily air pollution with MC as the index and death (24). However, by applying the same detection index and converting MC data into SAC analysis, we found a linear correlation between the SAC of particles in the ambient air and death data, indicating that SAC may be more suitable as an air exposure index (25). These studies suggest that MC alone cannot replace NC or SAC indicators.

Although we had selected the most common Chinese hotel kitchen in this study, it is still a case report. The sampling results of ultrafine particles were closely related to sampling location, distance, wind direction, air inlet direction, operation conditions, and protective measures (26, 27). The wind speed in the kitchen environment was relatively stable. Still, the movement of operators as well as equipment interfered with the wind speed and direction, leading to changes in the distribution of ultrafine particles. Therefore, this study recorded the activity events and meteorological conditions of the sampling process in detail. According to the characteristics of kitchen operation posts, the components of kitchen oil fumes (28) are complex, mainly including over 200 kinds of aldehydes, ketones, hydrocarbons, fatty acids, alcohols, aromatic compounds, esters, lactones, and heterocyclic compounds, most of which are toxic or even strong carcinogens (such as benzopyrene, and heterocyclic amines). The workers were tracked and sampled during a complete lunch cycle. The results showed that a slight change in the surrounding environment had different effects on different detection instruments, however, specific reasons need to be further discussed. Since the physical and chemical characteristics of ultrafine particles are different from those of general particles (29), eliminating the influence of background mixing and external interference in the workplace environment on the results is a problem that needs to be addressed in future research.

The exposure, hazard level, and risk ratios for this scenario given by the Nanotool model were all 0.75, which is high. The revealed high proportion of carcinogens in cooking smoke supports the results of high-risk levels obtained from control banding tools, and the results of NC, MC, SAC, and individual NC confirm the high exposure risk.

Epidemiological studies reported that cooking fumes contain many carcinogens and exposure to them increases cancer risk, which provides evidence for the high-risk nature of such air pollution. Controlling occupational hazard exposure is the primary method for protecting workers with high-risk exposure. According to NIOSH regulations, a series of controls, including elimination, substitution, engineering, administrative, and PPE, have been used to implement feasible and effective controls. For the restaurant investigated in this study, elimination, and substitution were not feasible, and instead, the best way to control kitchen fumes was to use engineering control. Results of the risk assessment showed that prevention and control measures should include local exhaust ventilation (LEV), indicating that the effectiveness of the existing LEV of the restaurant was insufficient, the air velocity at the capture point should be at least 1.2 m · s^−1^ (30). The reasons for this may include insufficient wind speed, the unreasonable position of the exhaust hood, and rising airflow in response to high temperatures. The following prevention and control measures should be added to the existing measures to protect workers in similar restaurants: (1) The capture efficiency of LEV needs to be improved. (2) A preventive maintenance plan should be formulated to ensure the effectiveness of engineering control measures. (3) NIOSH certified N95 or P100 filter mask respirators should be used, and regular inspection should be conducted to ensure that workers wear PPE correctly.

## Data availability statement

The raw data supporting the conclusions of this article will be made available by the authors, without undue reservation.

## Author contributions

ZZ contributed to the conceptualization, data curation, investigation, funding acquisition, supervision, and writing of the original draft. XG contributed to the investigation, data curation, methodology, funding acquisition, and formal analysis. YC contributed to the investigation and funding acquisition. HZ and YJ contributed to the conceptualization, funding acquisition, review, and editing of the manuscript. All authors contributed to the article and approved the submitted version.

## Funding

This study was supported by the Zhejiang Medical and Health Science and Technology Plan Project in 2021 (2021431152).

## Conflict of interest

The authors declare that the research was conducted in the absence of any commercial or financial relationships that could be construed as a potential conflict of interest.

## Publisher's note

All claims expressed in this article are solely those of the authors and do not necessarily represent those of their affiliated organizations, or those of the publisher, the editors and the reviewers. Any product that may be evaluated in this article, or claim that may be made by its manufacturer, is not guaranteed or endorsed by the publisher.

## Abbreviations

Abbreviations: NC: number concentration
MC: mass concentration
SAC: surface area concentration
NIOSH: National Institute for Occupational Safety and Health
PPE: personal protective equipment
LEV: local exhaust ventilation
LSD: least-significant difference.
